# Posttranslational modifications of AMPA receptors in synaptic function and memory

**DOI:** 10.1007/s00018-026-06297-9

**Published:** 2026-06-19

**Authors:** Cristina A. Muñoz de León-López, Irene Navarro-Lobato, Zafar U. Khan

**Affiliations:** 1 Laboratorio de Neurobiología, Centro de Investigaciones Medico Sanitarias (CIMES), Universidad de Málaga, Calle Marqués de Beccaria, 3, Campus Teatinos S/N, 29010 Malaga, Spain; 2 Department of Medicine, Faculty of Medicine, University of Malaga, Campus Teatinos S/N, Malaga, Spain; 3 CIBERNED, Institute of Health Carlos III, Madrid, Spain

**Keywords:** GluA1 subunit, GluA2 subunit, GluA3 subunit, GluA4 subunit, AMPA receptor tetramer, LTP and LTD, Homeostatic plasticity, Scaling up, Scaling down, Memory capacity, Spatial memory, Recognition memory, Fear memory renewal, Post-traumatic stress disorder

## Abstract

AMPA receptors, members of the ionotropic glutamate receptor family, are the principal mediators of fast excitatory synaptic transmission in the brain, and their trafficking to and from synapses is critical for neuronal communication. AMPA receptor trafficking is primarily regulated by posttranslational modifications. These receptors undergo diverse site-specific posttranslational modifications, including phosphorylation, palmitoylation, ubiquitination, S-nitrosylation, N-glycosylation, O-GlcNAcylation, and SUMOylation. Crosstalk among these modifications further adds to the complexity of AMPA receptor regulation. Accumulating evidence indicates that posttranslational modifications not only govern receptor trafficking and synaptic retention but also regulate synaptic strength during both Hebbian and non-Hebbian forms of synaptic plasticity, as well as memory function. Long-term potentiation induces site-specific modifications that promote synaptic incorporation of AMPA receptors, whereas long-term depression is associated with modifications that drive receptor internalization. Mutations affecting posttranslational modification sites disrupt AMPA receptor trafficking, impair synaptic plasticity, and alter memory function. Moreover, aberrant regulation of AMPA receptor posttranslational modifications has been linked to memory dysfunction during aging and in several neurological disorders. This review summarizes current understanding of how posttranslational modifications of AMPA receptors regulate synaptic plasticity, contribute to memory function, and are implicated in cognitive decline in aging and in disorders such as Alzheimer’s disease and schizophrenia.

## Introduction

Alpha-amino-3-hydroxy-5-methyl-4-isoxazolepropionic acid (AMPA) receptors are members of the ionotropic glutamate receptor family [65]. They mediate the majority of fast excitatory synaptic transmission in the brain [3] and play essential roles in synaptic plasticity and memory formation [25, 30]. AMPA receptors are composed of four subunits, GluA1, GluA2, GluA3, and GluA4 [2, 39]. These subunits assemble into homo- or heterotetrameric complexes that form ion channels at the synaptic membrane [5]. Upon glutamate binding, these receptor channels open to allow the influx of Na⁺ ions into neurons [65]. Additionally, AMPA receptor assemblies lacking the GluA2 subunit are permeable to Ca^2^⁺ ions [11, 63]. Because each AMPA receptor subunit contributes distinct properties to ion selectivity and channel kinetics, subunit heteromerization further diversifies receptor function.

AMPA receptors undergo continuous, dynamic trafficking to and from the synaptic membrane, cycling in and out on timescales ranging from seconds to minutes [11]. This regulated trafficking is a fundamental mechanism underlying Hebbian forms of synaptic plasticity. Long-term potentiation (LTP) is associated with the insertion of GluA1-containing AMPA receptors into the synaptic membrane, whereas long-term depression (LTD) involves their removal (Chater and Goda, 2014). Accordingly, blocking the synaptic incorporation of GluA1-containing AMPA receptors prevents LTP [78], while disruption of their endocytosis attenuates LTD [55, 64]. AMPA receptor trafficking is primarily regulated by posttranslational modifications [1, 6, 36]. Accumulating evidence indicates that subunit-specific posttranslational modifications not only govern receptor trafficking but also control synaptic retention, single-channel conductance, and ultimately synaptic strength [8, 15, 56].

## Types of posttranslational modifications in AMPA receptors

AMPA receptors are subject to diverse posttranslational modifications, such as phosphorylation, palmitoylation, ubiquitination, nitrosylation, glycosylation, and SUMOylation. These modifications are essential for the regulation of synaptic plasticity and associated behaviors. Among them, phosphorylation is the most extensively studied, and numerous phosphorylation sites have been identified across the different AMPA receptor subunits [15].

### Phosphorylation of AMPA receptors

AMPA receptor subunits are phosphorylated at multiple sites by diverse kinases, and these phosphorylation events play a critical role in regulating receptor trafficking and ion channel conductance [36].

#### GluA1 subunit

**Fig. 1:**
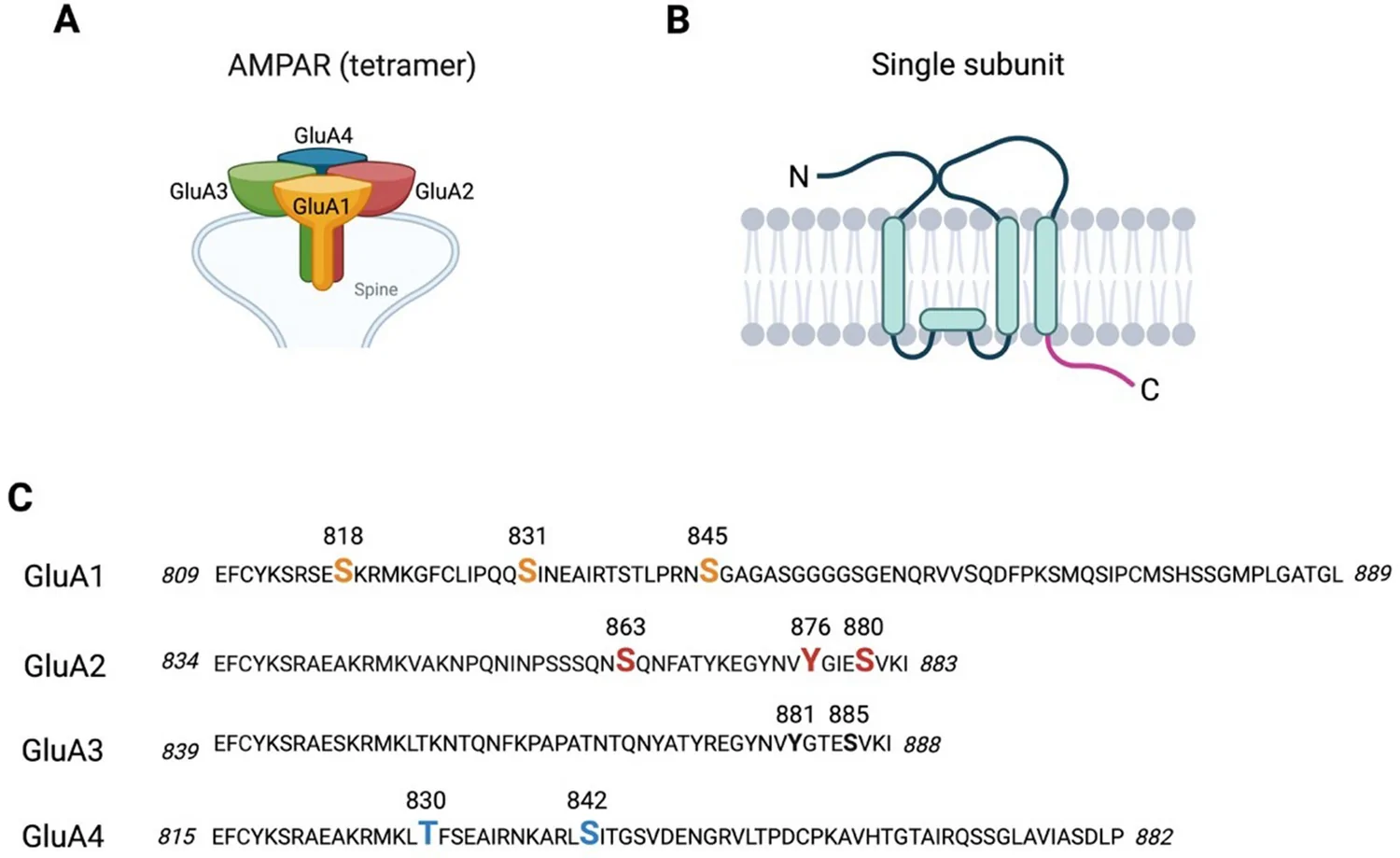
Phosphorylation sites in different subunits of AMPA receptors. (**A**) Schematic representation of tetrameric AMPA receptors composed of four subunits. (**B**) Illustration of a single AMPA receptor subunit embedded in the plasma membrane at the synaptic surface, highlighting the C-terminal region (shown in red), which is implicated in posttranslational modifications. (**C**) Amino acid residues (colored letters) and their corresponding positions (numbers) that have been demonstrated to undergo phosphorylation in the GluA1, GluA2, and GluA4 subunits. The GluA3 subunit contains analogous phosphorylation sites at Y881 and S885; however, direct evidence remains limited. N, N-terminal; C, C-terminal; S, serine; Y, tyrosine; T, threonine

Three major phosphorylation sites have been identified in the GluA1 subunit, all of which are localized to the carboxy-terminal (C-terminal) domain. These include serine 831 (S831), which is phosphorylated by both calcium-calmodulin-dependent protein kinase II (CaMKII) [51] and protein kinase C (PKC) [66], serine 845 (S845), which is phosphorylated by protein kinase A (PKA) [66], and serine 818 (S818), which is phosphorylated by PKC [4] (Fig. 1). Phosphorylation of S831 and S845 is required for trafficking of GluA1-containing AMPA receptors and for the expression of Hebbian plasticity, including LTP and LTD. However, these modifications alone are insufficient to drive stable synaptic incorporation of AMPA receptors [17, 29]. In contrast, phosphorylation of S818 is specifically required for LTP-driven incorporation of AMPA receptors into the postsynaptic membrane [36].

#### GluA2 subunit

The GluA2 subunit is phosphorylated within its C-terminal domain at serine 880 (S880) by PKC [13, 70], at tyrosine 876 (Y876) by Src family tyrosine kinases [27], and at serine 863 (S863) by PKC [52] (Fig. 1). Phosphorylation of both S880 and Y876 regulates AMPA receptor internalization during synaptic plasticity [12, 13, 27, 70]. In contrast, the functional significance of S863 phosphorylation remains unclear. Phosphorylation of GluA2 at either S880 or Y876 reduces its interaction with glutamate receptor–interacting protein 1 (GRIP1), while promoting the recruitment of protein interacting with C kinase 1 (PICK1) to the synaptic surface, thereby facilitating internalization of surface-localized AMPA receptors [13, 27, 36, 70].

#### GluA3 subunit

In contrast to GluA1 and GluA2, phosphorylation of the GluA3 subunit has been less extensively studied. GluA3 contains conserved residues analogous to GluA2 Y876 and S880 (GluA3 Y881 and S885), and these sites are likely phosphorylated, although direct functional evidence remains limited.

#### GluA4 subunit

GluA4 is phosphorylated at serine 842 (S842) within its C-terminal domain by PKA, PKC, and CaMKII, and is implicated in regulating receptor trafficking and synaptic insertion [68] (Fig. 1). A potential additional PKC site at threonine 830 (T830) has also been identified, though its functional role is less well defined [10, 36].

### Ubiquitination

**Fig. 2:**
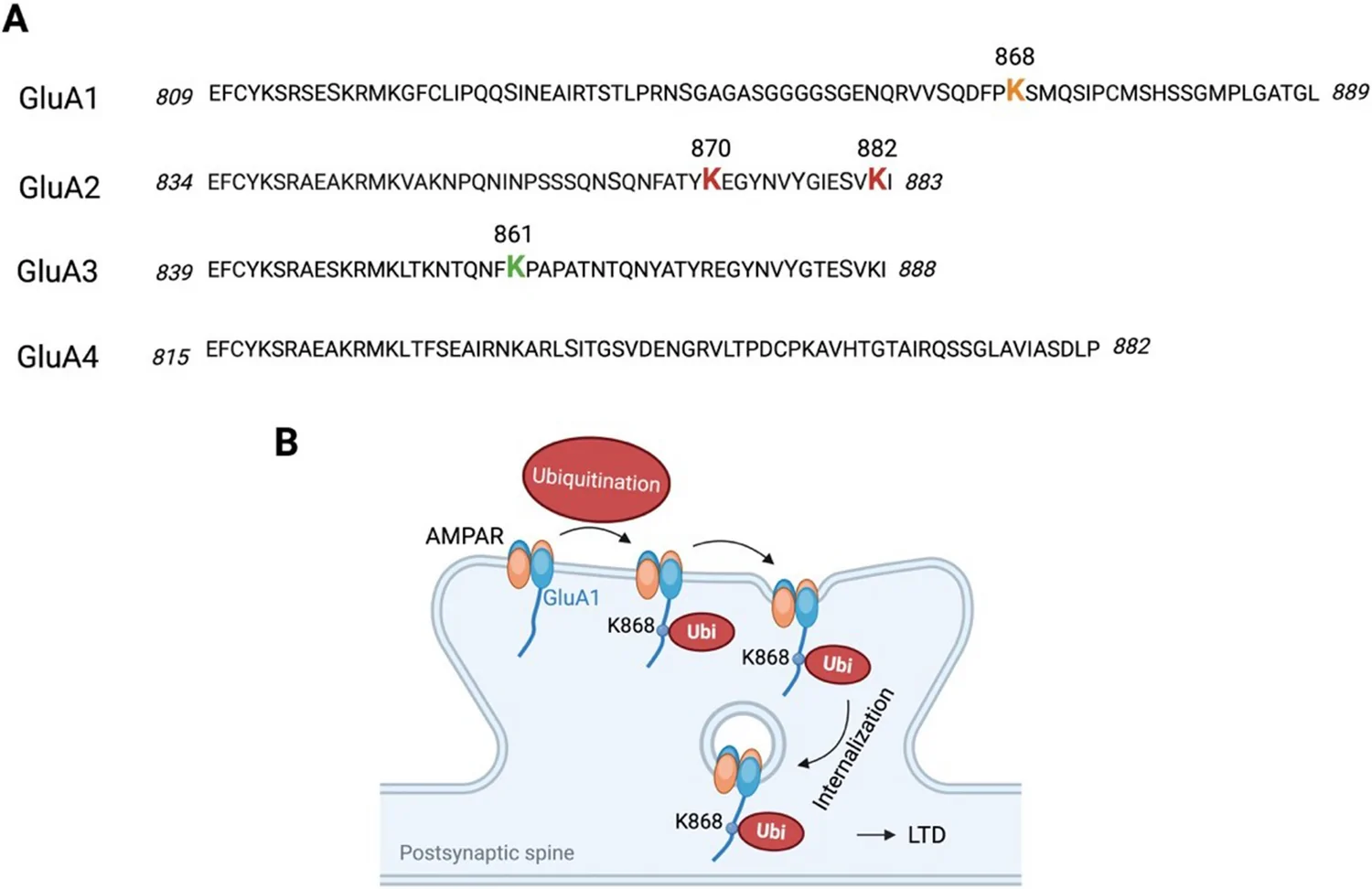
Ubiquitination of AMPA receptors. (**A**) Amino acid residues (colored letters) and their corresponding positions (numbers) that have been shown to undergo ubiquitination in the GluA1, GluA2, and GluA3 subunits. The ubiquitination site in the GluA4 subunit has not yet been identified. (**B**) Ubiquitination (Ubi) of the GluA1 subunit at lysine 868 (K868) promotes receptor internalization from the synaptic surface and facilitates the expression of LTD. K, lysine; AMPAR, AMPA receptor; LTD, long-term depression

Ubiquitination of AMPA receptors occurs at lysine residues within the C-terminal domain [81]. The GluA1 subunit is primarily ubiquitinated at lysine 868 (K868), whereas GluA2 is predominantly modified at lysines 870 (K870) and 882 (K882) [80] (Fig. 2). In addition, GluA3 undergoes ubiquitination at lysine 861 (K861) [92]. This modification is mediated by several E3 ubiquitin ligases, including neural precursor cell-expressed developmentally downregulated gene 4–1 (Nedd4-1), Nedd4-2, RNF167, and APC-Cdh1 [81]. Ubiquitination of GluA1 and GluA2 plays a critical role in the internalization of surface AMPA receptors [16, 62, 69], a process essential for long-term depression (LTD) [9, 36]. In contrast, ubiquitination of GluA3 primarily targets the receptor for degradation [92].

### Palmitoylation

**Fig. 3:**
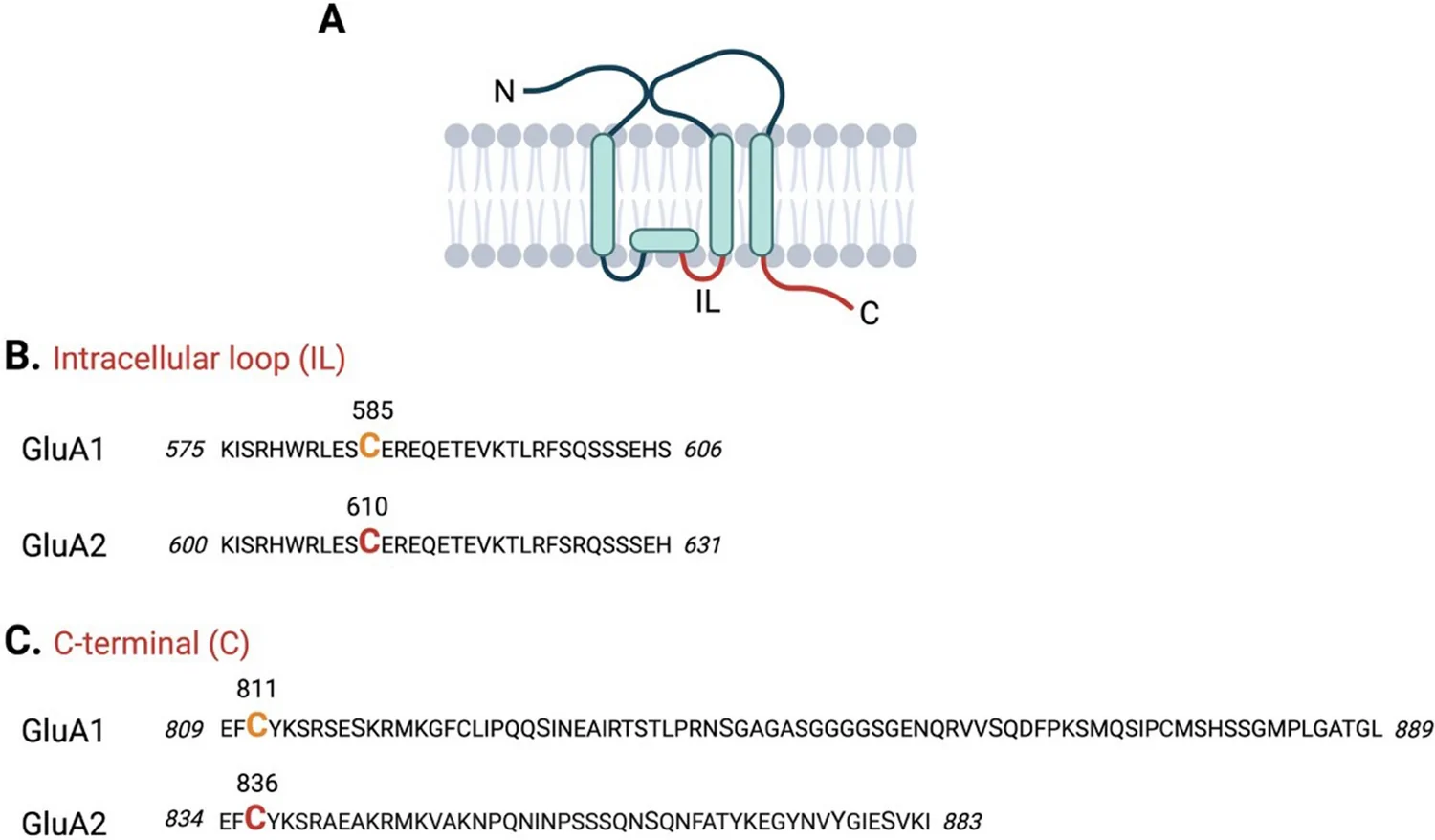
Palmitoylation sites in GluA1 and GluA2 subunits of AMPA receptors. (**A**) Schematic illustration highlighting the C-terminal and intracellular loop regions (shown in red) that are involved in posttranslational modifications. (**B** and **C**) Amino acid residues (colored letters) and their corresponding positions (numbers) that have been shown to undergo palmitoylation in the intracellular loop (B) and C-terminal (C) regions of the GluA1 and GluA2 subunits. N, N-terminal; C, cysteine residue

AMPA receptors are palmitoylated at two distinct cysteine residues, one within the intracellular loop between transmembrane domains 2 and 3, and another within the intracellular C-terminal region. Palmitoylation of GluA1 at cysteine 585 (C585) and GluA2 at cysteine 610 (C610) within the intracellular loop, mediated by palmitoyl acyltransferases, causes the accumulation of AMPA receptors in the Golgi apparatus [73], resulting in reduced receptor incorporation at the synaptic surface [28, 79] (Fig. 3). In contrast, palmitoylation at cysteine 811 (C811) in GluA1 and at cysteine 836 (C836) in GluA2 within the C-terminal domain does not alter basal synaptic surface levels of AMPA receptors. Instead, C-terminal palmitoylation of GluA1 disrupts its interaction with protein 4.1N [73], which normally stabilizes AMPA receptors at the synaptic surface, thereby facilitating agonist-induced AMPA receptor internalization [28, 73]. Consequently, C-terminal-palmitoylated AMPA receptors are more susceptible to ligand-induced internalization, whereas intracellular-loop-palmitoylated AMPA receptors retained in the Golgi are released upon agonist-mediated stimulation to travel toward the synaptic surface. Consistent with this, glutamate-mediated stimulation reduces AMPA receptor palmitoylation in the intracellular loop and promotes recruitment of additional receptors to the synaptic surface, contributing to synaptic plasticity [28, 36].

### S-nitrosylation

**Fig. 4:**
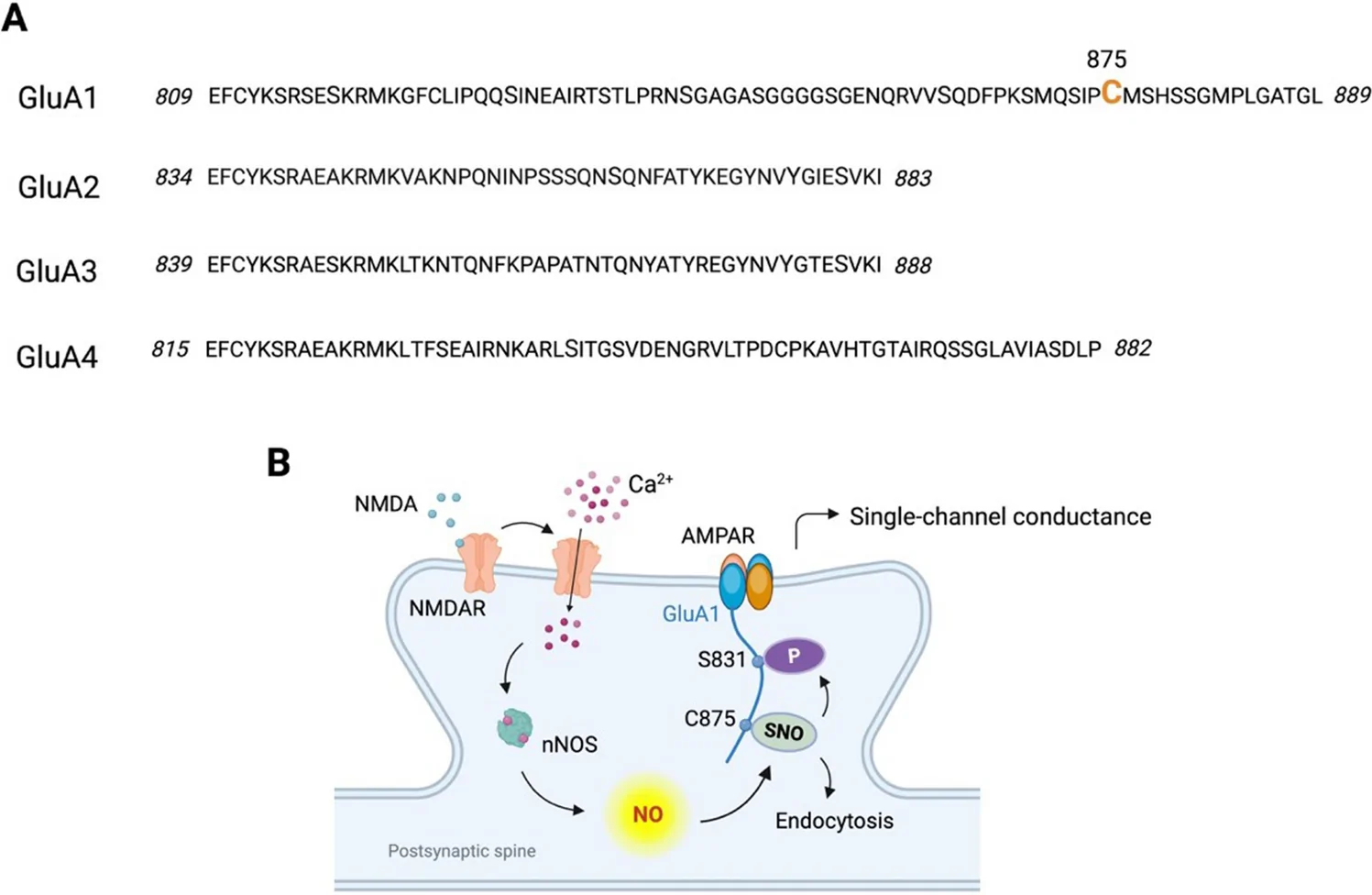
S-nitrosylation of AMPA receptors. (**A**) Amino acid residue (colored letter) and its corresponding position (number) that has been shown to undergo S-nitrosylation in the GluA1 subunit. S-nitrosylation sites in the GluA2, GluA3, and GluA4 subunits remain unidentified. (**B**) Activation of NMDA receptors (NMDARs) leads to Ca^2^⁺ influx, which activates neuronal nitric oxide synthase (nNOS) and promotes the production of nitric oxide (NO). NO subsequently induces S-nitrosylation (SNO) at cysteine 875 (C875) of the GluA1 subunit. S-nitrosylation at this site promotes AMPA receptor internalization and facilitates phosphorylation (P) at serine 831 (S831), thereby enhancing single-channel conductance. C, cysteine; NMDA, N-methyl-D-aspartate; AMPAR, AMPA receptor

S-nitrosylation of AMPA receptors has been demonstrated for the GluA1 subunit, whereas direct evidence for nitrosylation of GluA2, GluA3, and GluA4 remains limited. Following N-methyl-D-aspartate (NMDA) receptor activation, GluA1 undergoes S-nitrosylation at cysteine 875 (C875) by neuronal nitric oxide synthase (nNOS) within its C-terminal domain [71] (Fig. 4). Under conditions that favor receptor endocytosis, S-nitrosylation of GluA1 at C875 facilitates AMPA receptor internalization by increasing binding to adaptor protein complex 2 (AP2). However, S-nitrosylation of GluA1 at C875 can also modulate phosphorylation at S831 to enhance AMPA receptor single-channel conductance [71] (Fig. 4). Mutation of cysteine 875 to serine in GluA1 (C875S) abolishes the increase in single-channel conductance, a process that depends on phosphorylation of GluA1 at S831 [36, 51].

### N-glycosylation

**Fig. 5:**
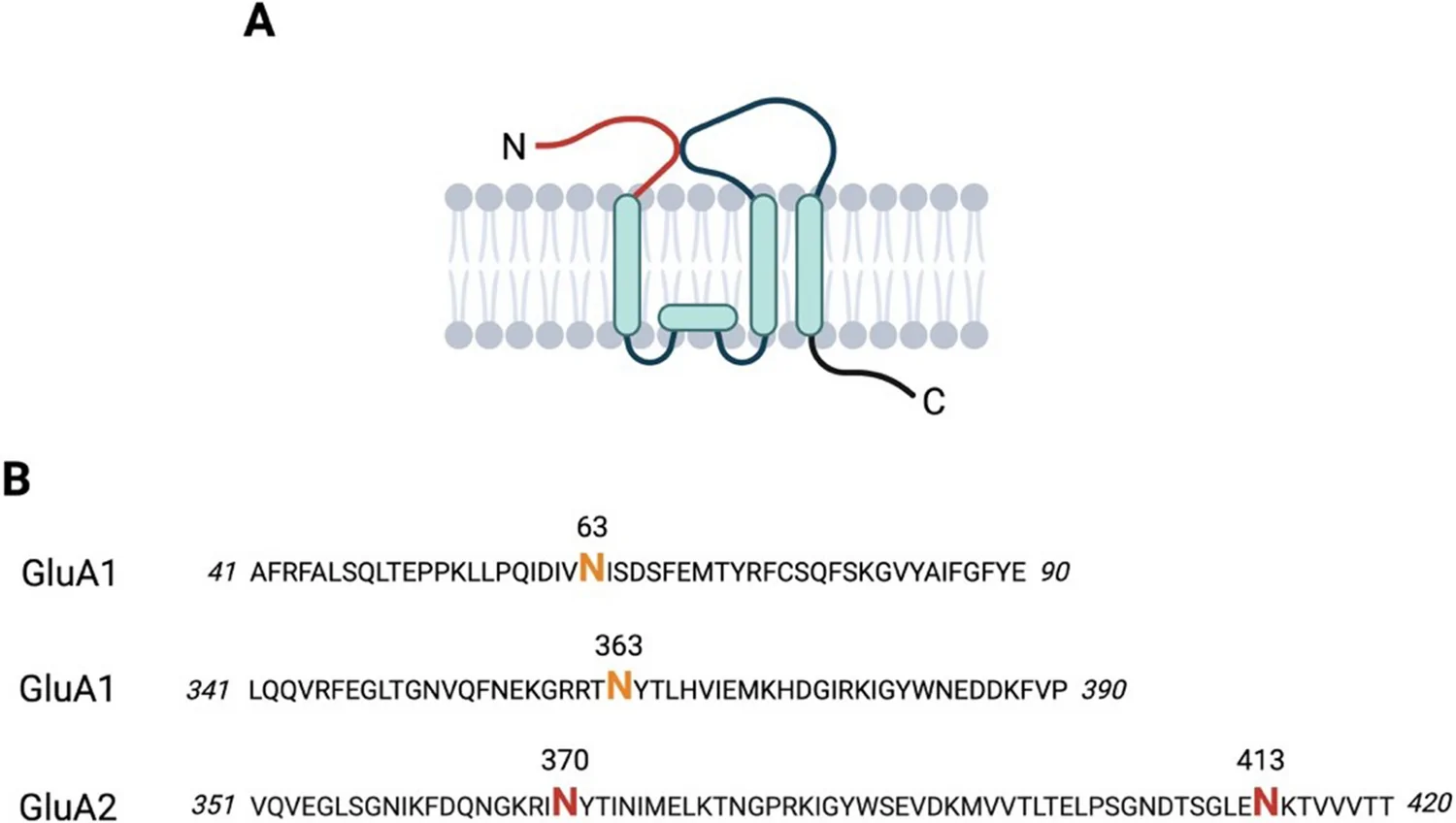
N-glycosylation sites in GluA1 and GluA2 subunits of AMPA receptors. (**A**) Schematic illustration highlighting the N-terminal region (shown in red), which is involved in posttranslational modifications. (**B**) Amino acid residues (colored letters) and their corresponding positions (numbers) that have been identified as N-glycosylation sites at two distinct positions within each of the GluA1 and GluA2 subunits. N, asparagine residue; C, C-terminal

AMPA receptor subunits undergo N-linked glycosylation at asparagine residues within their extracellular N-terminal domains, a process mediated by the oligosaccharyltransferase (OST) complex. In GluA1, N-glycosylation occurs at asparagines 63 (N63) and 363 (N363), whereas GluA2 is glycosylated at asparagines 370 (N370) and 413 (N413) [37, 54, 76] (Fig. 5). These modifications regulate tetramer assembly within the endoplasmic reticulum and promote forward trafficking of AMPA receptors to the cell surface. Disruption of N-glycosylation impairs receptor exit from the endoplasmic reticulum, resulting in altered synaptic localization and receptor function [54, 76].

### O-GlcNAcylation

Direct biochemical evidence of O-GlcNAcylation has been reported for the AMPA receptor GluA2 subunit, in which O-linked N-acetylglucosamine (O-GlcNAc) modification is associated with the regulation of synaptic plasticity and AMPA receptor trafficking; however, the precise O-GlcNAc modification sites remain undefined [31, 77]. In contrast, specific O-GlcNAc modifications in GluA1, GluA3, or GluA4 have not been conclusively identified. Nevertheless, global modulation of O-GlcNAc levels has been shown to influence AMPA receptor synaptic expression and function [23, 31, 41, 77].

### SUMOylation

Although SUMOylation has been shown to be required for increased GluA1 surface localization following glycine-induced LTP, direct SUMO conjugation of the GluA1 subunit has not been conclusively demonstrated [34]. Furthermore, to date, SUMOylation of the GluA2, GluA3, and GluA4 subunits has not been reported.

## Cross-talk between posttranslational modifications

**Fig. 6:**
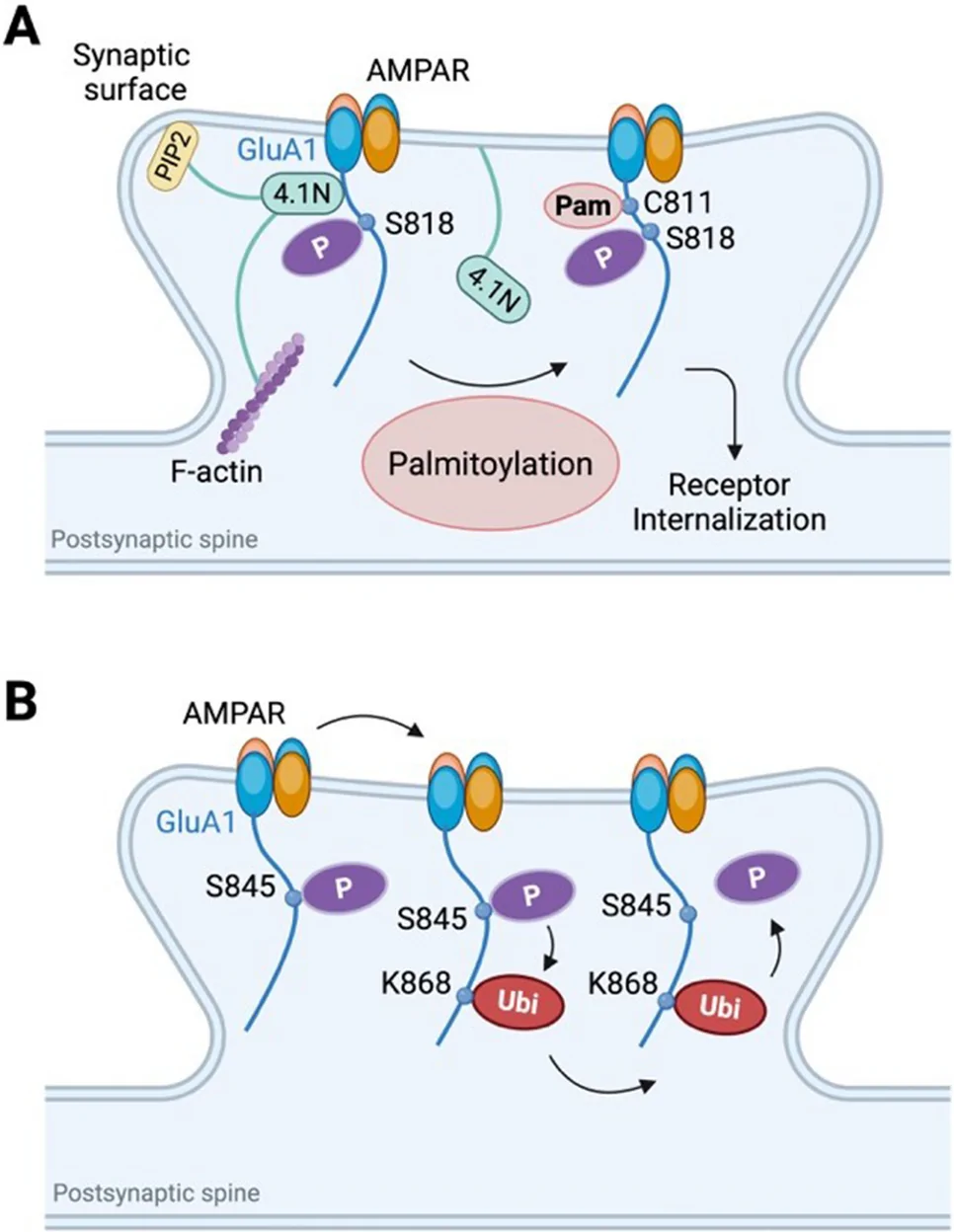
Crosstalk between phosphorylation, palmitoylation, and ubiquitination of the GluA1 subunit of AMPA receptors. (**A**) Schematic illustration showing that phosphorylation (P) at serine 818 (S818) enhances the association of GluA1 with 4.1N, which is linked to phosphatidylinositol 4,5-bisphosphate (PIP2) and F-actin, thereby stabilizing and retaining the receptor at the synaptic surface (left). In contrast, palmitoylation (Pam) at cysteine 811 (C811) in the GluA1 subunit counteracts this interaction, leading to receptor internalization (right). (**B**) Schematic illustration showing that phosphorylation (P) at serine 845 (S845) in the GluA1 subunit is associated with ubiquitination (Ubi) at lysine 868 (K868), which in turn contributes to reduced phosphorylation at S845. AMPAR, AMPA receptor

Different posttranslational modifications engage in functional crosstalk to regulate AMPA receptor trafficking and, consequently, receptor function. Within the C-terminal domain of GluA1, the absence of palmitoylation at cysteine 811 (C811) facilitates phosphorylation at serine 818 (S818) [46]. Phosphorylation at S818 promotes the interaction of GluA1 with protein 4.1N, thereby supporting synaptic retention of the receptor. In contrast, palmitoylation at C811 disrupts this interaction, leading to internalization of GluA1-containing AMPA receptors from the synaptic surface [46, 73] (Fig. 6A). In addition, a ubiquitin-deficient GluA1 mutant (K868R) exhibits enhanced phosphorylation at S845, a modification that is important for regulating synaptic surface incorporation of GluA1-containing AMPA receptor through recycling [21] (Fig. 6B). Conversely, mutation of the S845 phosphorylation site reduces GluA1 ubiquitination, suggesting a regulatory crosstalk between GluA1 phosphorylation and ubiquitination [21]. Furthermore, palmitoylation-deficient mutants of GluA2 (C610S) exhibit receptor instability and rapid degradation [82], indicating that palmitoylation of GluA2 at C610 is critical for receptor stability in the endoplasmic reticulum and may suppress ubiquitination and subsequent GluA2 degradation [48]. NMDA receptor–mediated S-nitrosylation of the GluA1 subunit at C875 promotes phosphorylation at S831 and enhances AMPA receptor single-channel conductance [71]. Mutation of C875 to serine (C875S) markedly reduces single-channel conductance, which is dependent on phosphorylation at S831 [71]. These findings suggest that S-nitrosylation of GluA1 at C875 can regulate S831 phosphorylation.

## AMPA receptor posttranslational modifications and synaptic plasticity

Posttranslational modifications are key regulators of AMPA receptor insertion, retention, and removal at the synaptic surface. Consequently, they provide a rapid and reversible mechanism for controlling both synaptic and biophysical properties of AMPA receptor–mediated transmission underlying Hebbian and non-Hebbian forms of synaptic plasticity. Through site-specific modifications, neuronal activity governs the expression of various forms of synaptic plasticity, including LTP and LTD. A summary of AMPA receptor site-specific posttranslational modifications and their effects on various forms of synaptic plasticity is provided in Table 1.

**Table 1:** Effect of AMPA receptor posttranslational modifications on synaptic plasticity

Plasticity type	AMPA receptor subunit	Posttranslational modification	Site of modification	Role in plasticity	References
LTP	GluA1	Phosphorylation	S831	+	[43, 85]
		Phosphorylation	S818	+	[15]
		Phosphorylation	S845	+	[64]
		Palmitoylation	C811	-	[35, 60]
	GluA4 (Postnatal)	Phosphorylation	S842	+	[49]
LTD	GluA1	Dephosphorylation	S845	+	[67]
		Ubiquitination	K868	+	[22]
		Palmitoylation	C811	-	[35, 60]
	GluA2	Phosphorylation	S880	+	[12]
		Phosphorylation	Y876	+	[27]
		O-GlcNAcylation	Not known	+	[77]
Homeostatic *Scaling up* *Scaling down*	GluA1	Phosphorylation	S845	+	[14, 72]
	GluA1 GluA2	Dephosphorylation	S845	+	[14]
		Phosphorylation	Y876	+	[86]

*LTP* long-term potentiation; *LTD* long-term depression; + direct role in synaptic plasticity; – no direct role in synaptic plasticity

### AMPA receptors phosphorylation in synaptic plasticity

#### GluA1 phosphorylation in LTP

**Fig. 7:**
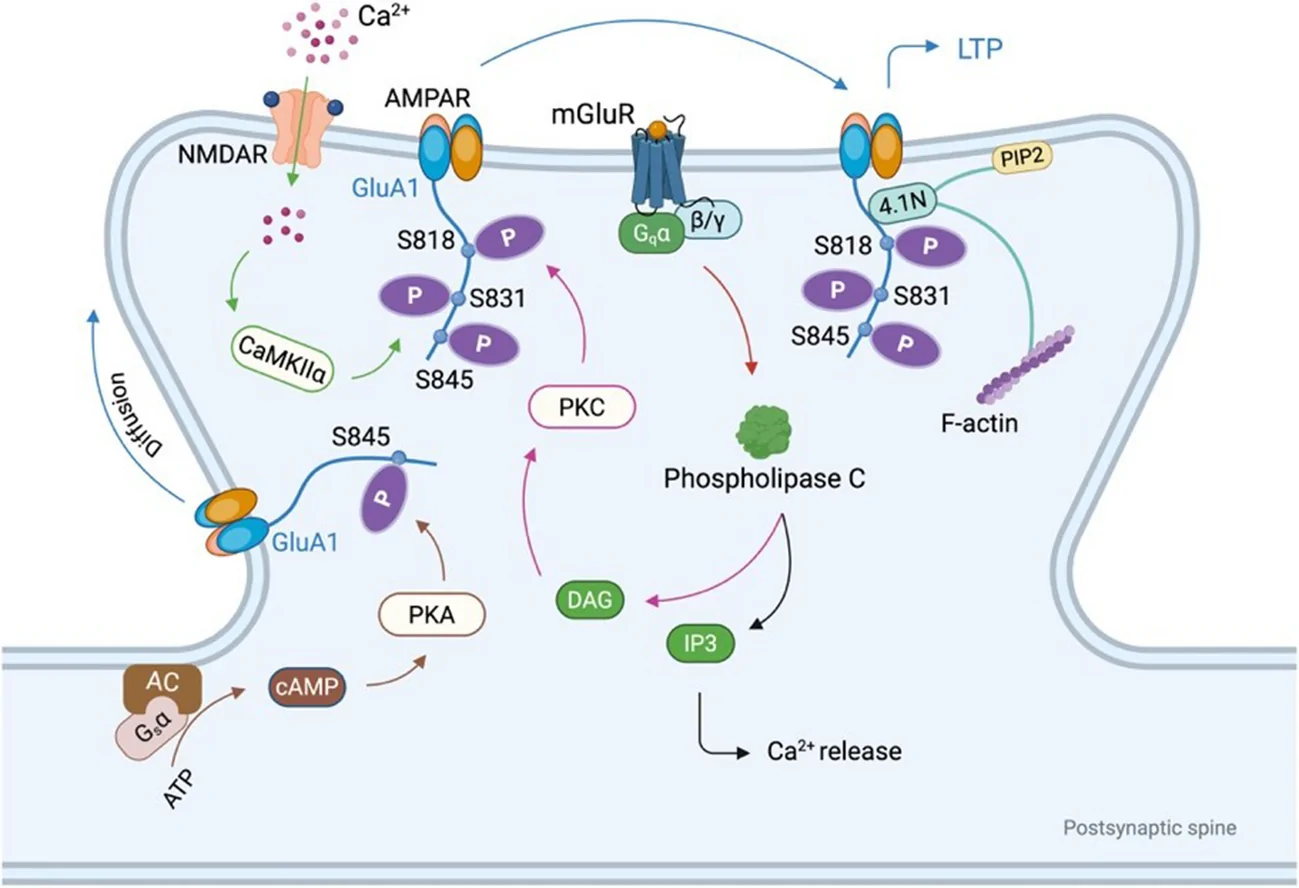
GluA1 phosphorylation in LTP expression. Adenylyl cyclase (AC) converts adenosine triphosphate (ATP) into cyclic AMP (cAMP), which activates protein kinase A (PKA). PKA phosphorylates (P) the GluA1 subunit at serine 845 (S845), promoting synaptic incorporation of AMPA receptors. Activation of NMDA receptors (NMDARs) permits Ca^2^⁺ influx, which activates Ca^2^⁺/calmodulin-dependent protein kinase II alpha (CaMKIIα). Activated CaMKIIα subsequently phosphorylates the GluA1 subunit at serine 831 (S831). Concomitantly, metabotropic glutamate receptor (mGluR)-mediated activation of phospholipase C (PLC) generates diacylglycerol (DAG), which activates protein kinase C (PKC). PKC then phosphorylates the GluA1 subunit at serine 818 (S818). Phosphorylation at this site promotes binding of protein 4.1N, thereby stabilizing AMPA receptors at the synaptic surface and supporting sustained LTP expression. Gsα, G protein alpha subunit; PIP2, phosphatidylinositol 4,5-bisphosphate; IP3, inositol 1,4,5-trisphosphate

Induction of LTP causes Ca^2^⁺-mediated activation of CaMKII, which phosphorylates GluA1 at S831 within the C-terminal domain [47, 85]. This modification enhances single-channel conductance and increases synaptic strength [43, 85] (Fig. 7). In contrast, phosphorylation of GluA1 at S818 by PKC is required for activity-dependent increase in synaptic levels of GluA1-containing AMPA receptors during LTP, largely through receptor stabilization at the postsynaptic membrane [15]. Phosphorylation at S818 promotes the interaction between GluA1 and protein 4.1N, thereby supporting synaptic retention of the receptor [46] (Fig. 7). Unlike S831, phosphorylation at S818 has not been shown to directly regulate single-channel conductance. Phosphorylation of GluA1 at S845 by PKA promotes synaptic incorporation of GluA1-containing AMPA receptors during LTP and increases channel open probability, resulting in enhanced AMPA receptor–mediated currents [64]. Transgenic mice carrying double knock-in mutations at S831 (S831A) and S845 (S845A) in GluA1 exhibit significant deficits in LTP, indicating that phosphorylation of GluA1 at these sites is critical for LTP expression [36, 42, 56].

#### GluA1 dephosphorylation and GluA2 phosphorylation in LTD

**Fig. 8:**
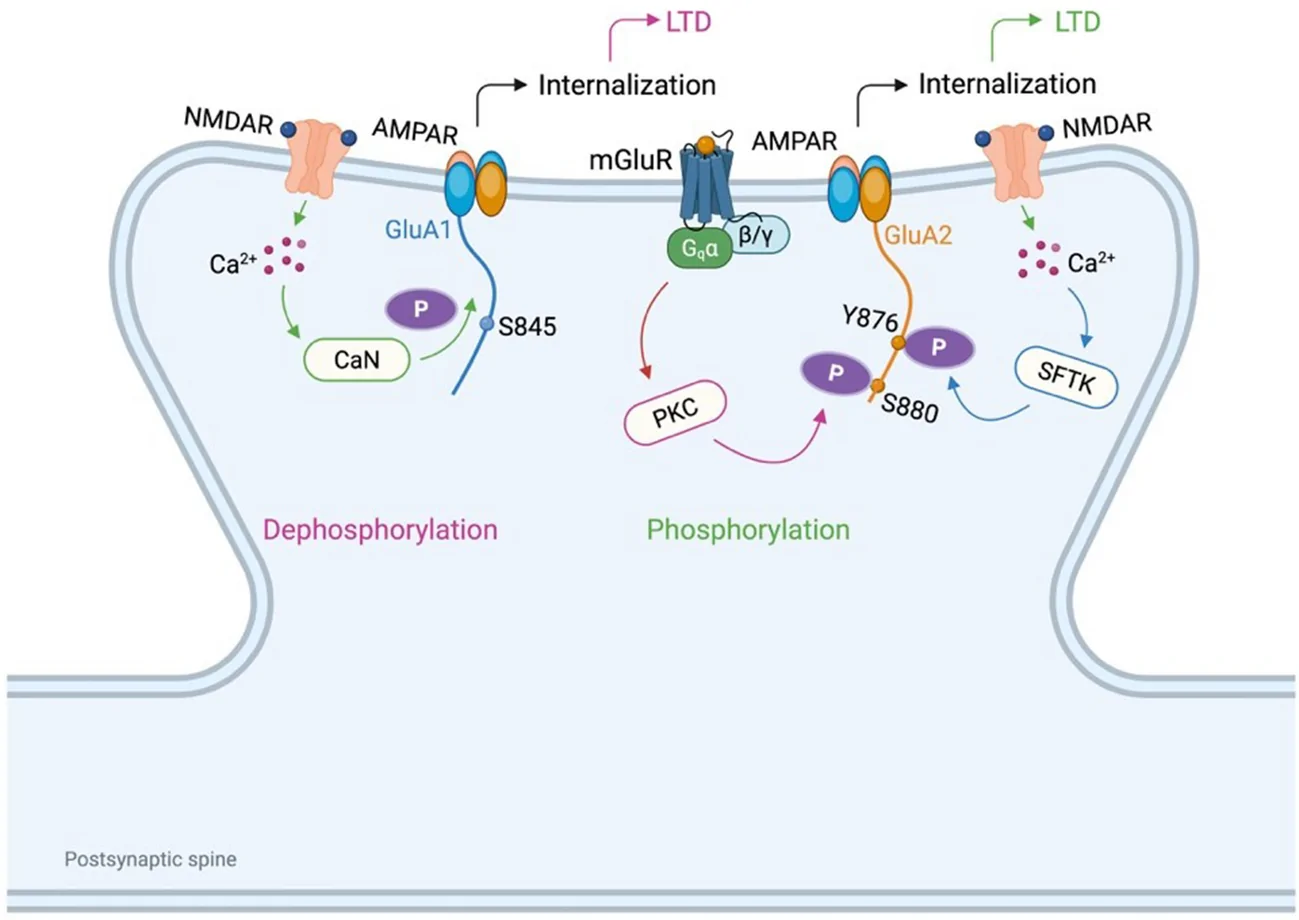
Dephosphorylation of GluA1 and phosphorylation of GluA2 in LTD expression. Ca^2^⁺ influx through NMDA receptors (NMDARs) activates calcineurin (CaN), a protein phosphatase. Activated CaN dephosphorylates the GluA1 subunit at serine 845 (S845), leading to internalization of AMPA receptors (AMPARs) and the expression of LTD. In contrast, phosphorylation of the GluA2 subunit at serine 880 (S880) by protein kinase C (PKC), activated via metabotropic glutamate receptor (mGluR) signaling, as well as phosphorylation at tyrosine 876 (Y876) by Src family tyrosine kinases (SFTKs), activated by Ca^2^⁺ influx through NMDARs, promotes receptor internalization and LTD expression. AMPAR, AMPA receptor

In contrast to LTP, which involves the insertion of GluA1-containing AMPA receptors at the synaptic surface, LTD is characterized by the internalization of AMPA receptors from the synaptic membrane (Chater and Goda, 2014). Induction of NMDA receptor–dependent LTD triggers dephosphorylation of GluA1 at S845 by the calcium-calmodulin-dependent phosphatase calcineurin, thereby facilitating internalization of GluA1-containing AMPA receptors from the synaptic surface [67] (Fig. 8). Blocking this dephosphorylation prevents receptor internalization and suppresses LTD [55, 67]. In contrast, metabotropic glutamate receptor 1 (mGluR1)–dependent LTD induces phosphorylation of the GluA2 subunit at S880 by PKC, which disrupts its interaction with GRIP1, a scaffold that stabilizes AMPA receptors at the synaptic surface [12, 24, 61]. The released GluA2 subsequently recruits PICK1, promoting receptor internalization [13, 89]. Moreover, both NMDA receptor– and AMPA receptor–dependent forms of LTD are associated with Src family tyrosine kinase–mediated phosphorylation of GluA2 at Y876, which, similar to phosphorylation at S880, disrupts its interaction with GRIP1 and promotes recruitment of PICK1 to facilitate AMPA receptor internalization [27, 36, 56].

#### GluA4 phosphorylation in LTP

GluA4 plays a critical role in synaptic plasticity during early postnatal development, a period when GluA4 is highly expressed [93]. In contrast, GluA4 expression is markedly reduced in the adult brain [93]. Induction of LTP at immature synapses triggers phosphorylation of GluA4 at serine 842 (S842), predominantly mediated by PKA, and this modification promotes the insertion of GluA4-containing AMPA receptors into the synaptic membrane [20, 49, 91]. In mature neurons, where GluA4 expression is low, LTP predominantly relies on GluA1-containing AMPA receptors, highlighting a developmental shift in subunit-specific mechanisms of synaptic potentiation [36, 56, 93].

#### GluA1 and GluA2 phosphorylation in homeostatic plasticity

Similar to LTP and LTD, homeostatic synaptic scaling fine-tunes synaptic strength by regulating the levels of AMPA receptors at the synaptic surface. Homeostatic scaling up is associated with phosphorylation of GluA1 at S845 [19, 40] and increased synaptic surface expression of GluA1-containing AMPA receptors [14, 19, 72]. This phosphorylation is facilitated by increased local availability of active PKA via anchoring to the scaffold protein AKAP5, along with concurrent suppression of calcineurin-mediated dephosphorylation [14]. In contrast, homeostatic scaling down is mediated either by reduced GluA1 phosphorylation at S845, caused by uncoupling of PKA from the AKAP5 scaffold and consequent suppression of PKA activity [14], or by Src family tyrosine kinase–mediated phosphorylation of the GluA2 subunit at Y876 [86]. Both downregulation of GluA1 phosphorylation at S845 and phosphorylation of GluA2 at Y876 facilitate AMPA receptor internalization from the synaptic surface [56].

### GluA1 ubiquitination in LTD

Ubiquitination of GluA1 at lysine 868 (K868) promotes internalization of surface AMPA receptors [16, 62], a process required for the expression of LTD [9]. Mice carrying a knock-in mutation at this ubiquitination site in GluA1 (K868R) exhibit deficits in both NMDA receptor– and mGlu receptor–dependent LTD, whereas LTP is enhanced [22]. These findings indicate that GluA1 ubiquitination is critical for LTD expression and that disruption of ubiquitination-dependent receptor internalization leads to synaptic surface accumulation of GluA1-containing AMPA receptors, resulting in enhanced LTP [36].

### GluA1 palmitoylation in LTD and LTP

Palmitoylation of GluA1 at C811 within its C-terminal domain inhibits the interaction between GluA1 and protein 4.1N, a cytoskeletal adaptor that stabilizes AMPA receptors at the synaptic membrane, thereby promoting activity-dependent internalization of GluA1-containing AMPA receptors [28]. In contrast, in the absence of palmitoylation at C811, enhanced phosphorylation of GluA1 at S818 promotes its interaction with protein 4.1N, leading to increased levels of synaptic surface GluA1-containing AMPA receptors [46]. Nevertheless, knock-in mice lacking the palmitoylation site at C811 (C811S) exhibit normal LTP and LTD, indicating that C811 palmitoylation is not essential for the induction of synaptic plasticity in vivo, possibly due to compensatory mechanisms [32, 33]. These findings suggest that while C811 palmitoylation dynamically regulates GluA1 trafficking, it is not strictly required for long-term synaptic plasticity [35, 60, 73].

### GluA1 S-nitrosylation in synaptic plasticity

NMDA receptor–dependent nitric oxide production via nNOS promotes S-nitrosylation of the GluA1 subunit at C875 [71]. This modification facilitates phosphorylation of GluA1 at S831, leading to enhanced AMPA receptor single-channel conductance [71]. Mutation of C875 to serine (C875S) significantly reduces S831 phosphorylation and attenuates the increase in single-channel conductance, indicating that S-nitrosylation at C875 is required for this effect [51]. In contrast, under conditions that favor receptor endocytosis, S-nitrosylation of GluA1 also promotes receptor internalization by enhancing its interaction with the adaptor protein complex 2 (AP2) [71]. Although increased conductance and receptor internalization are molecular features associated with early LTP and LTD, respectively [36], direct evidence linking AMPA receptor S-nitrosylation to long-term synaptic plasticity remains limited.

### GluA2 O-GlcNAcylation in LTD

Elevated O-GlcNAcylation induces an NMDA receptor–independent form of LTD at hippocampal CA3–CA1 synapses, which is mediated by O-GlcNAcylation of the GluA2 subunit [77]. This increase in O-GlcNAcylation also interferes with the induction of LTP. Consistently, enhanced O-GlcNAcylation suppresses excitatory synaptic transmission at Schaffer collateral–CA1 synapses by promoting the internalization of synaptic surface GluA2-containing AMPA receptors [31]. In contrast, inhibition of O-GlcNAcylation facilitates LTP at Schaffer collateral–CA1 synapses and promotes synaptic incorporation of AMPA receptors [38].

## AMPA receptor posttranslational modifications in memory function and dysfunction

Memory formation and retrieval depend on changes in synaptic strength that are critically mediated by AMPA receptor trafficking and function. Posttranslational modifications of AMPA receptors provide a molecular link between activity-dependent synaptic plasticity and memory processes by regulating receptor localization, stability, and signaling efficacy. Precise spatiotemporal control of these modifications is required for normal memory encoding and consolidation, whereas their dysregulation contributes to memory decline during aging and in neuropsychiatric and neurodegenerative disorders.

### Role in memory function

To date, relatively few studies have directly linked posttranslational modifications of AMPA receptors to memory functions, and among the available studies, most have focused on the GluA1 subunit. Evidence demonstrating the effects of site-specific modifications on various types of memory is summarized in Table 2.

**Table 2:** Effect of AMPA receptor posttranslational modifications on memory

AMPA receptor subunit	Posttranslational modification	Site of modification	Effect on memory	Type of memory	References
GluA1	Phosphorylation	S831	+	Memory capacity	[59]
	Phosphorylation	S845	+	Spatial, recognition, and fear memory. Memory capacity,	[18, 26, 59]
	Ubiquitination	K868	+	Spatial memory and cognitive flexibility	[22]
	Palmitoylation	C811	-	Spatial, recognition, and fear memory	[60, 75]
	S-nitrosylation	Not defined	+	Fear memory renewal	[74]
GluA2	O-GlcNAcylation	Not known	-	Object recognition and spatial memory	[77, 84]

+ effect on memory; - no effect on memory

#### GluA1 phosphorylation

Loss of phosphorylation of the GluA1 subunit at both S831 and S845 has been shown to impair memory capacity, defined as the ability to retain multiple object-related information simultaneously [59]. Phosphorylation of GluA1 at S845 is essential for spatial memory [18] as well as for recognition and contextual fear memory retrieval [26]. Additionally, experience-dependent tasks such as exploration of a novel environment elicit phosphorylation of GluA1 at S831 [7].

#### GluA1 ubiquitination

A direct association between GluA1 ubiquitination and memory has been demonstrated in GluA1 K868R ubiquitin-deficient knock-in mice, which exhibit impaired short-term spatial memory and cognitive flexibility, highlighting the importance of activity-dependent GluA1 ubiquitination in regulating memory processes [22]. While GluA2 ubiquitination is synaptic activity-dependent and this modification regulates AMPA receptor internalization [50], direct evidence for a role of GluA2 ubiquitination in memory function remains limited.

#### GluA1 palmitoylation

Increased palmitoylation of the GluA1 subunit impairs its localization to the plasma membrane and leads to deficits in object recognition and spatial memory [75]. In contrast, mice carrying a C-terminal palmitoylation-deficient mutation in GluA1 (C811S) exhibit prolonged contextual fear memory while maintaining intact sociability and spatial memory [60], indicating that GluA1 palmitoylation negatively regulates object recognition and spatial memory.

#### GluA2 O-GlcNAcylation

Elevated O-GlcNAcylation of the GluA2 subunit impairs object recognition memory [77]. Consistent with this finding, transgenic mice with increased global O-GlcNAcylation levels (Oga^ +/−) exhibit deficits in spatial memory [84]. Therefore, similar to GluA1 palmitoylation, GluA2 O-GlcNAcylation negatively affects both object recognition and spatial memory.

#### GluA1 S-nitrosylation

Fear memory renewal is defined as the return of a conditioned fear response after extinction when the subject is re-exposed to the conditioned stimulus. It has been shown that S-nitrosylation is required for fear renewal [74], and that fear renewal critically depends on phosphorylation of the GluA1 subunit at S831 [44]. Inhibition of nNOS, the enzyme responsible for activity-dependent S-nitrosylation, attenuates fear renewal [74], suggesting that S-nitrosylation supports fear memory renewal, likely by increasing GluA1 phosphorylation.

### Role in memory dysfunction and memory deficits

There is relatively limited literature directly addressing the role of posttranslational modifications of AMPA receptors in memory dysfunction and cognitive deficits. Among the available studies, the majority have focused on phosphorylation of the GluA1 subunit, highlighting its prominent role in regulating AMPA receptor function under pathological conditions. Evidence linking GluA1 phosphorylation to various forms of memory impairment is summarized in Table 3.

**Table 3:** Impact of AMPA receptor posttranslational modifications on memory impairment

Condition/disease	AMPA receptor subunit	Posttranslational modification	Site of modification	References
Deficit of fear memory extinction (PTSD)	GluA1	Phosphorylation	S845	[58]
Enhancement of fear memory renewal (PTSD)	GluA1	Phosphorylation	S831	[44]
Memory decline during aging	GluA1	Phosphorylation	S831 S845	[83]
Memory deficits in Alzheimer´s disease	GluA1	Phosphorylation	S845	[45, 53, 90]
Memory deficits in schizophrenia	GluA1	Phosphorylation	S845	[87, 88]

#### GluA1 phosphorylation in deficit of fear memory extinction

Deficits in fear memory extinction are a hallmark of post-traumatic stress disorder (PTSD). Acute restraint stress induces anxiety-like behavior and impairs fear extinction, an effect that is mediated by increased phosphorylation of the GluA1 subunit at S845 [58]. This stress-induced increase in GluA1 S845 phosphorylation is associated with enhanced synaptic surface incorporation of GluA1-containing AMPA receptors and impaired fear memory extinction.

#### GluA1 phosphorylation in enhancement of fear memory renewal

In contrast to deficits in fear memory extinction, enhancement of fear renewal, defined as the context-specific relapse of conditioned fear following extinction, also contributes to PTSD. Phosphorylation of the GluA1 subunit at S831 is required for fear memory renewal, and increased phosphorylation at this site enhances fear relapse in stressed animals [44]. It has been suggested that GluA1 phosphorylation at S831 during fear renewal may be facilitated by increased S-nitrosylation of the GluA1 subunit, as inhibition of S-nitrosylation abolishes fear renewal [74].

#### GluA1 phosphorylation in memory decline during aging

The phosphorylation levels of the GluA1 subunit at both S831 and S845 are reduced in cognitively impaired aged rats, and this decrease is accompanied by diminished synaptic surface incorporation of GluA1-containing AMPA receptors [83].

#### GluA1 phosphorylation in memory deficits in Alzheimer´s disease

Accumulating evidence implicates dysregulated GluA1 phosphorylation in Alzheimer’s disease (AD)–related cognitive decline. In line with this, reduced phosphorylation of the GluA1 subunit at S845 has been associated with spatial memory deficits in the APP_Sw,InS_ transgenic mouse model of AD [53]. Consistently, increasing GluA1 phosphorylation at S845 through overexpression of the 85 kDa isoform of ribosomal protein S6 kinase 1 (p85S6K), a serine–threonine kinase, ameliorates spatial and object recognition memory deficits in the APP/PS1 mouse model of AD [45]. Furthermore, enhancement of GluA1 phosphorylation at S845 also rescues spatial memory impairments induced by β-amyloid treatment [57, 90].

#### GluA1 phosphorylation in memory deficits in schizophrenia

Cognitive deficits are a core feature of schizophrenia. Restoration of GluA1 phosphorylation at S845 in a rat model of schizophrenia has been shown to reverse both fear and spatial memory deficits [87]. In addition, treatment with olanzapine, an atypical antipsychotic, rescues spatial memory deficits in a rat model of schizophrenia by upregulating GluA1 phosphorylation at S845 [88].

#### A shared link between phosphorylation of the GluA1 subunit at S845 site and memory deficits

Emerging evidence suggests that impaired phosphorylation of the GluA1 subunit at S845 represents a convergent molecular mechanism underlying memory deficits across aging and several neurological disorders. Phosphorylation at this site, primarily mediated by PKA, promotes synaptic surface incorporation of AMPA receptors and facilitates synaptic potentiation associated with memory formation. In aged animals, reduced phosphorylation of GluA1 at S845 is associated with decreased synaptic incorporation of AMPA receptors and cognitive impairment. Similarly, in Alzheimer’s disease models, impaired S845 phosphorylation correlates with deficits in spatial and recognition memory, whereas restoration of phosphorylation at this site ameliorates memory dysfunction. A comparable reduction in GluA1 S845 phosphorylation has also been reported in schizophrenia models, where pharmacological or molecular interventions that restore phosphorylation improve both fear-associated and spatial memory deficits. Together, these findings suggest that dysregulation of GluA1 S845 phosphorylation may constitute a shared pathological mechanism contributing to impaired synaptic plasticity and memory dysfunction across multiple brain disorders.

## Integration of posttranslational modifications in AMPA receptor trafficking, synaptic plasticity, and memory

**Fig. 9:**
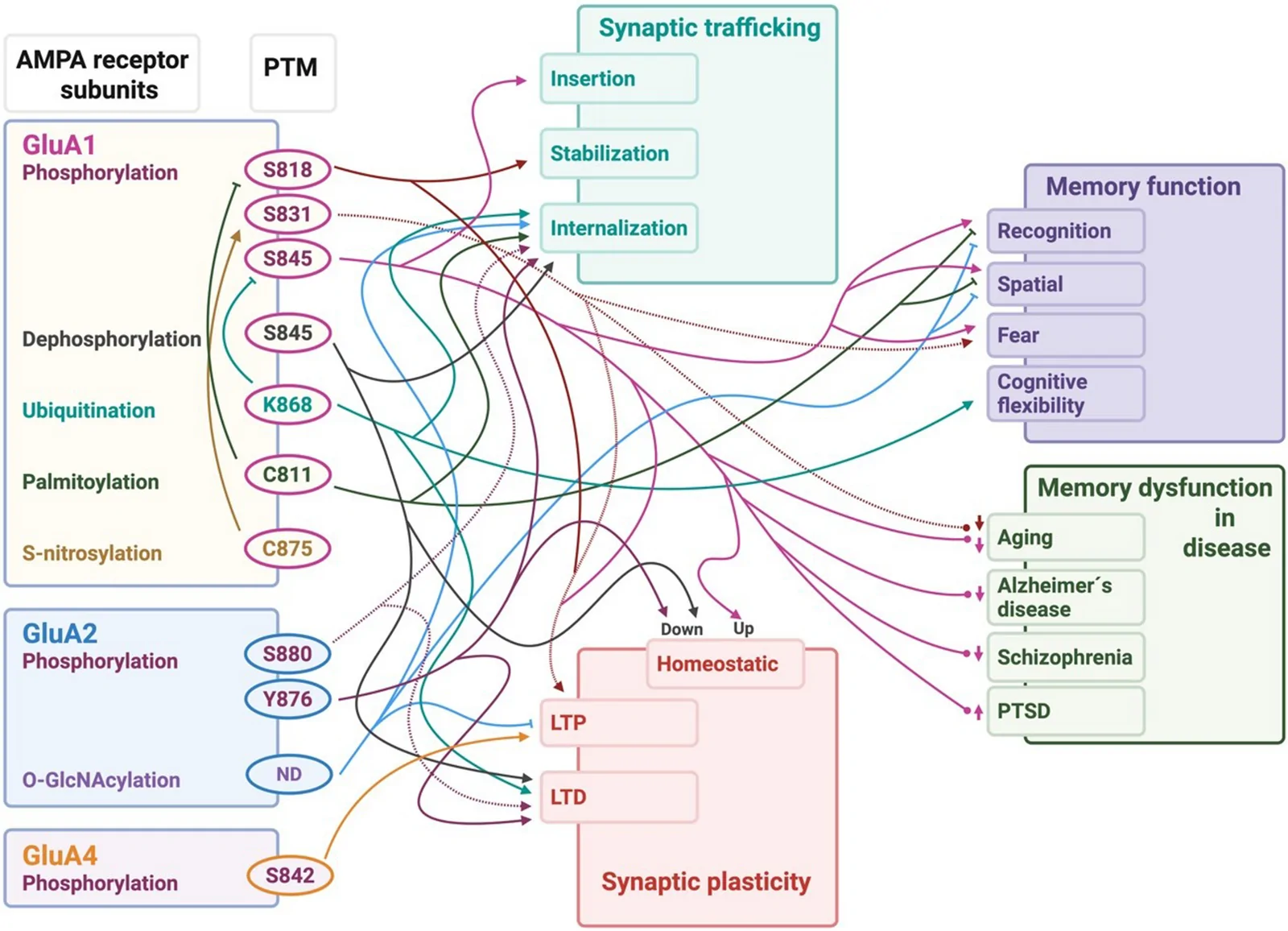
Integrated effects of AMPA receptor posttranslational modifications on receptor trafficking, synaptic plasticity, and memory. Schematic illustration summarizing key posttranslational modifications across AMPA receptor subunits and their effects on receptor trafficking, both Hebbian and non-Hebbian forms of synaptic plasticity, and memory function and dysfunction. Among these modifications, phosphorylation of the GluA1 subunit at S845 emerges as a central regulator promoting synaptic insertion of AMPA receptors and LTP induction, and is critically involved in memory processes. In addition, dysregulation of phosphorylation at this site in the GluA1 subunit is associated with memory deficits in aging and neurological disorders. In contrast, O-GlcNAcylation of the GluA2 subunit regulates internalization of synaptic AMPA receptors and is associated with reduced synaptic potentiation and impaired memory performance. Crosstalk among posttranslational modifications further refines these processes, as S-nitrosylation of the GluA1 subunit enhances phosphorylation at S831, whereas palmitoylation and ubiquitination negatively regulate GluA1 phosphorylation. In the illustration, interconnecting lines ending with arrowheads indicate positive effects or upregulation, whereas lines ending with perpendicular lines indicate negative effects or downregulation. Lines ending with filled circles within the *Memory dysfunction in disease* panel denote changes in phosphorylation levels (increase or decrease), as indicated by adjacent arrows. PTM, posttranslational modification; ND, not determined; Down, scaling down; Up, scaling up

Evidence suggests that AMPA receptor-mediated function is not governed by individual posttranslational modifications in isolation but rather by their coordinated and dynamic interplay. Multiple site-specific modifications, including phosphorylation, palmitoylation, ubiquitination, and S-nitrosylation, converge on shared molecular processes such as synaptic surface incorporation, retention, and internalization. Through this coordinated regulation, posttranslational modifications fine-tune the number and functional properties of AMPA receptors at synapses, thereby shaping synaptic strength (Fig. 9).

At the level of synaptic plasticity, these modifications act in a highly integrated manner to control the bidirectional trafficking of AMPA receptors during LTP and LTD. For instance, phosphorylation of the GluA1 subunit at S845 and S831 promotes receptor insertion and enhances channel conductance during LTP, whereas phosphorylation of GluA2 at S880, along with ubiquitination and palmitoylation of GluA1, facilitates receptor internalization during LTD (Fig. 9). Importantly, crosstalk among these modifications further refines these processes, as exemplified by the interaction between phosphorylation and ubiquitination or the reciprocal regulation between palmitoylation and phosphorylation in controlling receptor stability at the synaptic surface.

These molecular and synaptic events ultimately translate into functional outcomes at the level of neural circuits and behavior. By regulating AMPA receptor trafficking and synaptic efficacy, posttranslational modifications directly influence memory functions. For example, phosphorylation of GluA1 at S845 and S-nitrosylation facilitate synaptic incorporation of AMPA receptors and enhance single-channel conductance, mechanisms that contribute to LTP induction and the promotion of spatial, recognition, and fear memory (Fig. 9). These observations suggest that memory functions are dynamically regulated by the density of surface AMPA receptors, where increased surface expression facilitates multiple forms of memory.

Dysregulation of posttranslational modifications is implicated in PTSD as well as in memory impairments associated with aging and neurological disorders. Specifically, increased phosphorylation of GluA1 at S845 is associated with enhanced synaptic surface incorporation of AMPA receptors, a mechanism linked to PTSD-like phenotypes. Conversely, reduced phosphorylation at this site is observed in memory decline associated with aging, Alzheimer’s disease, and schizophrenia (Fig. 6). These contrasting observations indicate that precise regulation of S845 phosphorylation is critical for cognitive function, as its disruption contributes to memory dysfunction.

Together, these findings highlight that AMPA receptor posttranslational modifications operate as an integrated regulatory system linking molecular signaling to synaptic plasticity and memory outcomes. A comprehensive understanding of this coordinated network will be essential for elucidating the mechanisms underlying cognitive function and for identifying potential therapeutic targets in memory-related disorders.

## Concluding remarks

Posttranslational modifications of AMPA receptors constitute a central regulatory mechanism governing receptor trafficking to and from the synaptic surface, as well as the functional properties of excitatory synapses. However, because most studies of these modifications have focused primarily on the GluA1 subunit, the contributions of other AMPA receptor subunits remain less well understood. Additionally, there is a substantial gap in the identification and characterization of posttranslational modification sites across AMPA receptor subunits. For instance, phosphorylation sites have been identified in the GluA1, GluA2, and GluA4 subunits, whereas ubiquitination sites have been reported in GluA1, GluA2, and GluA3. In contrast, palmitoylation sites have thus far been identified only in GluA1 and GluA2, and S-nitrosylation has been demonstrated only in the GluA1 subunit. Furthermore, although O-GlcNAcylation has been reported in the GluA2 subunit, the precise modification sites remain unidentified, similar to SUMOylation, for which modification sites have not yet been characterized in any AMPA receptor subunit.

Among the various posttranslational modifications, phosphorylation is the most extensively studied. Multiple phosphorylation sites within the GluA1, GluA2, and GluA4 subunits have been shown to regulate receptor trafficking and synaptic function, whereas phosphorylation sites within the GluA3 subunit remain largely unexplored. Nevertheless, the presence of analogous phosphorylation sites in the GluA2 subunit (Y876 and S880) and the GluA3 subunit (Y881 and S885) suggests that GluA3 may also participate in regulating synaptic AMPA receptor internalization. Similar to phosphorylation, ubiquitination of the GluA1 and GluA2 subunits plays a critical role in AMPA receptor trafficking. In contrast, palmitoylation of GluA1 and GluA2 within the C-terminal domain primarily regulates receptor stability and retention at the synaptic surface rather than directly controlling receptor trafficking. The roles of ubiquitination and palmitoylation in other AMPA receptor subunits, as well as those of other posttranslational modifications in regulating AMPA receptor trafficking and synaptic function, are only beginning to emerge, and the currently available literature remains comparatively limited.

Current evidence suggests that site-specific posttranslational modifications of AMPA receptors enable precise control of synaptic strength underlying both Hebbian and non-Hebbian forms of synaptic plasticity. These modifications not only shape synaptic function but also play critical roles in memory formation and consolidation. Synaptic incorporation of AMPA receptors during LTP is primarily regulated by phosphorylation of the GluA1 subunit at S845 and S831, whereas receptor internalization during LTD is mediated through phosphorylation of the GluA2 subunit at S880 and Y876, as well as through ubiquitination (K868) and palmitoylation (C811) of GluA1 and O-GlcNAcylation of GluA2. In contrast, non-Hebbian homeostatic plasticity is regulated by phosphorylation of the GluA1 subunit at S845 to facilitate synaptic scaling up, whereas scaling down is mediated through phosphorylation of the GluA2 subunit at Y876. Crosstalk among posttranslational modifications also appears to play an important role in regulating AMPA receptor phosphorylation and associated synaptic functions. For example, S-nitrosylation of the GluA1 subunit at C875 enhances phosphorylation at S831 and increases AMPA receptor single-channel conductance. However, studies examining crosstalk among posttranslational modifications have thus far been largely limited to the GluA1 subunit, and whether similar mechanisms operate in other AMPA receptor subunits remains unexplored.

Among all posttranslational modifications, phosphorylation of the GluA1 subunit appears to play a particularly central role, being involved not only in synaptic insertion of AMPA receptors and the expression of both Hebbian and non-Hebbian forms of synaptic plasticity, but also in memory function. In contrast, O-GlcNAcylation of the GluA2 subunit regulates internalization of synaptic AMPA receptors and is associated with reduced synaptic potentiation and impaired memory performance. In particular, phosphorylation of GluA1 at S845 facilitates memory performance, and dysregulation of phosphorylation at this site has been implicated in memory decline associated with aging and neurological disorders such as Alzheimer’s disease and schizophrenia. Studies across these neuropathological conditions suggest that impaired phosphorylation of GluA1 at S845 may represent a convergent mechanism underlying memory dysfunction.

Despite significant progress, important questions remain regarding the subunit-specific roles of these modifications in regulating receptor trafficking, synaptic properties, circuit dynamics, and behavior. Future studies integrating advanced biochemical, imaging, electrophysiological, and genetic approaches will be essential to further elucidate how these modifications coordinate synaptic and cognitive functions. A deeper understanding of these mechanisms may provide novel therapeutic targets for memory-related disorders.

## Data Availability

Data sharing not applicable to this article as no datasets were generated or analyzed during the current study.
